# In Search of the Global East: Thinking between North and South

**DOI:** 10.1080/14650045.2018.1477757

**Published:** 2018-10-02

**Authors:** Martin Müller

**Affiliations:** Department of Geography and Sustainability, University of Lausanne, Lausanne, Switzerland; Center for Global Urbanism, Ural Federal University, Ekaterinburg, Russia

## Abstract

Carving up the world into Global North and Global South has become an established way of thinking about global difference since the end of the Cold War. This binary, however, erases what this paper calls the Global East – those countries and societies that occupy an interstitial position between North and South. This paper problematises the geopolitics of knowledge that has resulted in the exclusion of the Global East, not just from the Global North and South, but from notions of globality in general. It argues that we need to adopt a strategic essentialism to recover the Global East for scholarship. To that end, it traces the global relations of IKEA’s bevelled drinking glass to demonstrate the urgency of rethinking the Global East at the heart of global connections, rather than separate from them. Thinking of such a Global East as a liminal space complicates the notions of North and South towards more inclusive but also more uncertain theorising.

## Introduction: Losing the East

Picture the ‘Global North’ for a moment. Like most others, you will think of countries in North America and Western Europe, perhaps also Japan and Australia – the rich states and large metropolitan centres. And the Global South? Latin America and Africa come to mind, much of Asia, too. Places where people do not enjoy the same privilege as in the North. The world thus conjured before our inner eye appears complete, but it is far from. On the contrary, the binary of North and South creates a black hole: all those societies that fall somewhere between North and South – too rich to be in the South, too poor to be in the North. And this is no small black hole: it encompasses those societies that took part in what was the most momentous global experiment of the twentieth century: to create communism.^1^ This ‘Global East’, as this paper shall call it, is suspended somewhere in the shadows between the Global North and the Global South, not quite belonging to either. This paper seeks to retrieve it from there.

The distinction between a richer, powerful Global North and a poorer, less-powerful Global South is perhaps the most influential way of categorising the world and thinking about global difference today. That distinction has not just become a staple of academic research, spawning several journals, numerous research centres and hundreds of books that carry the Global South in the title. It has also started to enter the standard vocabulary of scholars, activists and, increasingly, policy debates. In climate change talks, for example, the North–South divide has become a prominent moniker for framing political differences over how to address global warming. The millennial rediscovery of poverty as a challenge by the United Nations Millennium Development Goals gave further currency to the North–South divide.

Rather than mere geographical descriptors, the Global North and South today signify primarily a political and epistemological project: a turn away from the language of developmentalism and teleological progress that characterised the attitudes of the Global North towards the South for decades and continues to characterise it today; a re-orientation of knowledge production from the universalism and euro-centrism in the North and a valorisation of a multiplicity of knowing practices as found in the Global South; and a political inspiration for the reconfiguration of global politics to give a voice to more marginal nations (Dirlik 2007; Mignolo 2011). As such, the Global South is part and parcel of the postcolonial project of making the subaltern speak (Spivak 1988a).

The fall of most communist regimes, the so-called Second World, between 1989 and 1992 did not challenge the North–South distinction; quite the opposite, it vindicated it. At Fukuyama’s (1992) end of history, the division between rich and poor, and thus between North and South, endured, whereas that between capitalism and communism vanished. With the evaporation of the communist Other, the ideological East–West division evaporated too. ‘With the disintegration of the Soviet Union, the Second World categorization became less useful. … The time was ripe for a new, simplifying categorization. The First World became the North and the Third World became the South’ (Reuveny and Thompson 2007, 557). But what about the Second World?

Rather than joining the North or the South, the East has fallen between the cracks. I use ‘East’ here as a shorthand not so much for a geographical region, but for an epistemic space – a liminal space in-between North and South. While I make sense of this East through the experience of the former Second World, the notion of the East as a global East is not limited to that experience but may, and indeed should, encompass other liminal societies. The demise of the Second World’s political project – communism – wiped the East off the global map, any distinctiveness of more than 70 years of communist rule erased. The East is too rich to be a proper part of the South, but too poor to be a part of the North. It is too powerful to be periphery, but too weak to be the centre. Power relationships run every which way. The East includes both colonisers and colonies, aggressors and victims; some countries were both at the same time (Tlostanova 2008). In other words, the East is slippery, hard to categorise.

In the global circulation of signs, the East is not nearly as legible as the Global South, where colonialism has created shared languages, institutions, knowledge systems and social bonds. Uganda is more easily knowable in the global centres of media and scholarship than Ukraine, Chile is more familiar than Czechia and Laos is closer than Latvia. Vargas Llosa, García Márquez and Coetzee have a ring of instant recognition, whereas Aleksievich, Müller and Szymborska sound outlandish. All six are recent laureates of the Nobel Prize in Literature.

The East therefore experiences a dual exclusion. For one thing, it is not considered part of the Global South. Vijay Prashad’s (2013) *The Poorer Nations*, one of the definitive histories of the Global South, wears its geographical allegiance on the cover sleeve: the map of the Global South depicted there includes Turkey, Argentina and Chile, but cuts off Kyrgyzstan, Moldova and Ukraine, for example – all of them quite a bit poorer. Comprehensive volumes on the Global South skip the Global East. *An Everyday Geography of the Global South* (Rigg 2007) features 90 case studies in 36 countries, but not one in the Global East. The *Handbook on Cities in the Global South* at least acknowledges that ‘much of Eurasia is ignored’ (Parnell and Oldfield 2014, 3). *Institutions of the Global South* (Braveboy-Wagner 2009) limits itself to Asia, Africa and Latin America. Those silences are all the more worrying since most scholars insist on a fluid notion of the Global South, without hard boundaries (Dirlik 2007; Roy and Crane 2015). Still, that notion does not seem fluid enough to include even parts of the former Second World.

But the East is also separate from the Global North. In writing about the world’s global cities and centres, the heartlands of democracy and of market capitalism, the East is a silent bystander. Countries in the East may be on the way northward, but at the same time seem stuck in eternal transition towards an elusive modernity. Object of the North American and European *mission civilisatrice*, the East is defined through its backwardness, a persistent marker of Eastern Europe for centuries (Kovačević 2008; Neumann 1999; Todorova 1997; Wolff 1994). It has served as the Other against which Western Europe has long narrated its own civilisation and progress.

This paper seeks to take one step in the global geopolitics of knowledge towards putting the East back on the map of knowledge production. It does so in thinking of it as a Global East – different from, yet connected to, the rest of the world; equal, not inferior, to the North and South. That is not just an important epistemic move for people who live in the Global East, valorising the distinctiveness and connectedness of their experience, and for scholars who work on the Global East, who are often at a loss as to how to position their subject in global scholarship that partitions the world into North and South.

It is even more a crucial move for theorising. For in an age of theorising from outside Euro-America (Bhabha 1994; Chakrabarty 2007; Mbembe 2000), good theorising, much less global theorising, cannot happen without an understanding and a profound appreciation of the diversity and interconnectedness of social realities that it purports to refer to. Thinking of a Global East is perhaps even more important for the Global North and the Global South, since taking the Global East seriously cannot happen without unsettling certainties of rich and poor, powerful and powerless, that we have perhaps grown too comfortable with. Recovering the Global East means thinking from the interstices of the North and the South – not just for the East, but also for the North and the South.

This article seeks to create the conditions for thinking of the Global East through a quadruple move. First, it analyses ‘Eastness’ as a predicament of being not so much on the margins as in the interstices between North and South. It therefore underscores Eastness as a liminal condition of in-betweenness – not-quite North, not-quite-South – rather than as a geographical location. This in-betweenness is the cause for meeting with ignorance both in the North and in the South. Second, it demonstrates how the East has remained unknowable, because it is outside the circuits and conduits of Western knowledge architecture. Third, it argues for the necessity of a strategic essentialism of the East that emphasises the unity in difference of the interstitial position of the East and seeks to recover the East as a political project. Finally, it shows how the East is and indeed has to be a Global East, by thinking of it as in the middle of global relations rather than cut off from them. It thus affirms the openness of the Global East against attempts, from the inside and outside, to wall it off.

## The Predicament of Eastness: Not-quite-North, Not-quite-South

When I teach on the Global East, my students are puzzled as to how I could possibly be interested in such a dull place. Brazil is sexy, Kenya cool, China dynamic – the East, however, is boring. I was both relieved and crestfallen to learn that I am not alone. In a review of the literary peculiarities of the East, Mikanowski (2017, n.p.) notes: ‘I know a distinguished scholar of the region, a historian who teaches a regular course on Eastern European history, who told me that every year he has to answer questions from his students about whether people actually love and laugh in this “grey place”’. I suppose I would rather teach on the ‘dark continent’, and at least garner the compassion of do-gooders, than on a ‘grey place’ that evokes no emotion at all – the terra incognita of the world, where Macedonia, Moldova, Montenegro and Molvanîa^2^ blend into an amorphous mass.

The predicament of the East marks a dual exclusion: from the entitled Global North and from the marginalised Global South. But it is not pure Otherness. It is rather a semi-alterity (Tlostanova 2017), a demi-orientalism (Wolff 1994). The East is different but similar, Other but not quite. As much a ‘grey zone’ of indeterminacy (Knudsen and Frederiksen 2015) as it is a grey place. The Global North, often in the guise of ‘Europe’, serves as the teleological horizon against which the East becomes a not-quite-North. Countries may join the European Union, but the subtle distinctions of habitus of Eurocrats in the corridors of power in Brussels continue to mark the difference between the East and the West (Kuus 2014) as does the racism that Eastern European immigrants experience in Western Europe (Nowicka 2017). People may partake of European consumption, such as in the ubiquitous *evroremont* (Sgibnev 2015), but never quite become fully European. This ‘Eastness’, as some scholars have called it (Kuus 2007; Zarycki 2014), has been a marker of the East for decades, if not centuries, notwithstanding EU accession, decades of economic growth, widespread privatisation and democratisation.

In this regard, the East shares much with the postcolonial condition of being in the ‘waiting room of history’ (Chakrabarty 2007, 8), striving for a modernity in which it may participate, but only at the discretion and grace of Europe, extending its *mission civilisatrice* and everything that comes with it to the ‘Wild East’ (Gille 2016; Horvat and Štiks 2012; Melegh 2006). For if the East were ever to arrive in Europe, ‘everything is [already] anticipated, thought out, demonstrated, made the most of’, to quote Fanon’s (2008, 91) famous phrase.^3^ What holds the East together is then not so much political unity, common economic ties or cultural traditions (Hann et al. 2016), no longer the common experience of socialism (Müller 2019), but rather a shared feeling of simultaneous difference *and* resemblance to an amorphous Europe.

One could also, in a more political economic inclination, read the East through Immanuel Wallerstein’s (1979) three-tier world-systems theory.^4^ But the picture becomes a little more muddled here. While for Wallerstein (1976) the socialist states belonged to the semi-peripheral countries, this picture has changed since the socialist implosion. Some Central Asian, South Caucasus and Southeast European states today plausibly count as peripheral, whereas most EU countries in Eastern Europe are economically growing more and more into the core. This, at least, is the observation of several sources that attempted to apply Wallerstein’s categories today (Babones and Babcicky 2011; Bradshaw 2001; Knox, Agnew and McCarthy 2014, 22). Those states that remain semi-peripheral, such as Russia or Romania, share the same, quite heterogeneous category of other semi-peripheral states like Greece, Chile, Botswana and Vietnam. A world-systems analysis therefore represents a splintering picture of the East as partly North, partly South and partly in-between.

Although the East, as demi-Other of the West, resembles the South’s postcolonial condition, it is not included in the South’s fight for emancipation. Just as it is not quite North, it neither is quite South. In the push for de-colonial knowledge and theorising from the South, the East gets no mention: it is not that it is lumped in with Latin America, Asia and Africa. No, it simply is not part of this project. Little does that surprise if we consider a standard definition of North and South:

The North-South divide is broadly considered a socio-economic and political divide. Generally, definitions of the Global North include the United States, Canada, developed parts of Europe, and East Asia. The Global South is made up of Africa, Latin America, and developing Asia including the Middle East (from Wikipedia, quoted in Mignolo 2014a, n.p.).

This definition encircles the Global East. So do attempts to theorise from the South. Raewyn Connell’s (2007) seminal *Southern Theory* locates the South in Africa, Latin America, India and Iran (exactly the regions mentioned in the definition above). Jean and John Comaroff’s (2011) *Theory from the South* looks at Africa and Ananya Roy’s (2009) *New Geographies of Theory* at India.

The absence of the East in the project of the Global South is striking, though perhaps not surprising. It has, again, to do with its in-between status – in social, economic and political terms. Comaroff and Comaroff (2011, 46) puzzle: ‘On which side [of North and South], for example, do the countries of the former USSR fall?’. As the former Second World, the societies of the East did not organise at venues such as the Bandung conference for a third way between capitalism and socialism. When the socialist camp collapsed during the events from 1989 to 1992, it looked as though, with the transition to capitalism, the East was going to join the North. Yet, even after more than 25 years of that collapse, that transition remains unfinished, both in terms of achieving the level of wealth in the North and in terms of building institutions of market capitalism. The East ended up with a hybrid of socialist legacies, neoliberal capitalism and informal and patrimonial practices.

Colonialism also marks the East, although in a different way from the South. Perhaps the most distinctive feature of the South (Dirlik 2007), also in terms of its importance for theorising, colonialism runs no straightforward way in the East. Many parts of the East have seen successive waves of colonisation, by the Ottoman Empire, the Austro-Hungarian Empire and the Russian Empire and the Soviet Union, with rather different systems of domination. The Western imposition of market reforms in the 1990s (Boycko, Shleifer and Vishny 1995) has added another relationship of domination to the pre-existing ones.

If the colonised is defined in relation to the coloniser (Fanon 1952), then the East has multiple identities. Some countries were both colonisers and colonies. Madina Tlostanova (2008, 2011) problematises this interstitial position for the case of Russia, cast into a dual role of both colonial empire (both during and after the Soviet Union) and subaltern Other (of Europe) – a subaltern empire, as she calls it. As such, the East sits uneasily between the mostly postcolonial societies of the South and the centres of power of the North. That turns the relationship coloniser/colonised in the East into a multi-layered one, where there is no clear metropole. If you are Bulgarian, is Istanbul, Moscow, Brussels or New York your metropole?

Finally, unlike in the South, people have not found in the East a cause for compassion, global activism or a source of alternatives to neoliberalism, environmental destruction, power politics and rampant nationalism. The former heart of Ronald Reagan’s ‘evil empire’, it lacks the moral high ground of the subaltern and disenfranchised that propels the Global South – Euromaidan and Rose, Tulip and Orange Revolutions notwithstanding. The wretched of the Earth, struggling for emancipation and self-determination, do not seem to reside in the East. Many people in the Global East are white and both perpetrators and victims of racism but also victims of racism, such as Polish immigrants in Britain (Nowicka 2017). ‘Russians and Eastern Europeans have become after 1989 the off-white blacks of the new global world – looking and behaving too similar to the same, yet remaining essentially other’ (Tlostanova 2017, 8). Katherine Verdery (2002, 20), in a powerful turn of phrase, once wrote that it was unclear who would be the Frantz Fanon of the post-socialist East. A tough question to which I would reply: There can be no Frantz Fanon of the East. After all, who would s/he speak to and with what right?

So the East is inferior, but not inferior enough. It is kind of subaltern, but not really. It is not rich, but neither is it poor. It has some elements of European modernity, but lacks others: too different to be included in the North, too European to be included in the South. Most societies of Eastern Europe and the former Soviet Union are embroiled in this interstitial relation of not quite North and not quite South. They may be members of the EU, even high-income countries, but still do not quite belong to the club. Just think of Poland. Or conversely, they may be poor and former colonies, such as Tajikistan or the Russian Caucasus, but are still not counted among the South. They are, at best, a ‘secondary South’ (Tlostanova 2011). Attempts to work towards a dialogue between the South of the Global East, such as the colonies of Soviet Russia, and the Global South are few and far between (Chari and Verdery 2009; Karkov 2015; Tlostanova 2011, 2015b)

It is that liminality that makes debates on the Global North and Global South pass by the East: not out of spite, but because the East does not fit the frame through which we think the global. So this in-betweenness does not turn the East into something like Homi Bhabha’s (1994) fertile third space, a trading zone between cultures and meanings. On the contrary, the East seems stuck in stasis, whereas the rest of the world has moved on to be enveloped in a net of global connections and mobilities. Writes Mikanowski:

Many times, I’ve fallen into pockets of Eastern Europe far west of the Oder–Trieste line. It’s happened to me below highway overpasses, in line at the DMV [Department of Motor Vehicles in the United States], and in the waiting rooms of neglected bus stations. I’ve always thought that Proustian moments were a completely false construct, a literary conceit, but I’ll be damned if I haven’t been caught by the smell of tired dirt covering a bathroom in the basement of one of Berkeley’s physics labs and, in an instant, been transported to the stairwell of my grandmother’s Warsaw apartment building, with its mixture of stale urine and tired dirt and old mop water, unsweetened by soap (Mikanowski 2017).

Mikanowski here captures Eastness as a feeling of forsakenness and of disconnection from the world. Highway overpasses, waiting rooms of neglected bus stations, basements – globality happens elsewhere. Eastness is an inert condition, as though fallen out of time and space.

This condition of being stuck in time manifests itself in how we refer to the geographical area of today’s East, always indexing the past – post-socialist, ex-Soviet, former USSR, old Eastern bloc, former Second World – as though, after almost 30 years, the communist East had not found its way into the present yet. Bestsellers such as Aleksievich’s Nobel Prize-winning *Second-Hand Time* or Orland Figes’s *Just Send Me Word* recall the Soviet experience. The bestselling books about current affairs are about the putative new cold war. One of the principal academic journals on the East describes itself as ‘focusing on the history and current political, social and economic affairs of the countries of the *former “communist bloc*”’ (Europe-Asia Studies 2018).

Included neither in the North nor in the South, stuck in stasis, the East has disappeared from ‘the global’ at large. Try to find a significant role of the East in debates on, say, global urbanism, global business or global mobilities. The point here is not so much that the East gets little mention, although that is often true as well. It is more that the East is not thought of as part of global connections (Rogers 2010). It does not partake of ‘the global’ – of global flows of images and ideas, people and policies. If the ‘East’ exists, it does not deserve the modifier ‘global’. One could be forgiven to believe that the iron curtain had never fallen.

## Outside the Western Knowledge Architecture

But the iron curtain is gone. A self-confident re-inscription of the East is overdue, not least to unsettle the binary of North and South. Claiming a voice for the East in academic debates can counter, or at least challenge, the dominant cultural circuits of knowledge production (Buchowski 2004; Timár 2004)^5^ and parallel attempts at a self-isolation of the East (Funk 2017). It can attempt to recast the hemispheric ‘geopolitics of knowledge’ (Mignolo 2002) increasingly organised into North and South.

Part of the political impetus of recovering the East is to reclaim the great diversity that so often gets written out and reduced to a caricature of a monotonous ‘grey place’. A diversity that is not just ethnic (although it is that as well), but political, cultural and economic – and that belies the homogenising moniker as ‘former Eastern bloc’, a favourite of news commenters still today. From the poster child of European reform policies that is Estonia via the dictatorship in Belarus to the global ambitions of Kazakhstan. From the former imperial core that is Russia via conflict-torn Ukraine to the centrifugal peripheries of Slovenia. That diversity, and its effects on people’s lives, is perhaps best encapsulated in the joke about the old man who says he was born in Austria-Hungary, went to school in Czechoslovakia, married in Hungary, worked most of his life in the Soviet Union, and retired in Ukraine. ‘Travelled a lot, then?’ asks his interviewer. ‘No, I never moved from Mukachevo.’

The larger point at stake here is that the East does not fit easily into existing architectures of knowledge in a mostly Anglophone world (Tlostanova 2015a). English does not travel far as a *lingua franca*, Western colonialism has not established shared institutions or family bonds and the after-effects of the iron curtain, which had complicated the forging of research collaborations, can still be felt. Émigré intellectuals and scholars from the Global South – Stuart Hall, Gayatri Spivak, Edward Said, Achille Mbembe, Aimé Césaire – often ended up in the colonial centres in Britain, France or the United States, inserting themselves into Anglophone or Francophone circuits of knowledge production. Yet, if Eastern scholars went, as they often did, to Moscow, the centre of the Soviet empire, they spoke to a limited audience and, after the collapse of the Soviet Union, faced a growing linguistic isolation and declining global reach. For aspiring intellectuals in the East, French and German, not English, the language of market capitalism, were the foreign languages of choice. That influenced in what circuits knowledge from the East and about the East could travel. Ironically, remaining outside British and French colonialism limited the chances of the East to be heard.

Another dynamic at play in the absence of voices from the East in global debate is the blow that the collapse of socialism dealt to scholarship in the East. The impact of that blow is still felt today. Not just did funding vanish almost overnight, but academics found themselves in a situation where the idea of what makes a good scholar had changed radically. Many left academia and their countries in order to survive and those who stayed had to (and sometimes still have to) work side jobs to make money, especially in those disciplines like the humanities and social sciences whose work was thought to be of little practical value. Academic work offered no future and lacked a viable income, so few young scholars could enter academia in the 1990s and 2000s to weigh in to debates. Instead, they went to study and work abroad (Ushkalov and Malakha 2010). Against this background, it is not surprising ‘that the removal of restrictions on publishing, and the social transformations themselves, in Central and Eastern Europe had not led to an explosion of new home-grown analyses of communist and postcommunist reality’ (Outhwaite and Ray 2005, 12) How could it? The socialist collapse had all but wrecked research in the East and the situation has just started to change in the past few years.

## Towards a Strategic Essentialism of the East

Considering these odds, the political project of reclaiming a voice for the East becomes even more important. In so doing, I think we should keep the term ‘East’, not shying away from confronting its old connotations of backwardness and otherness. For terms such as ‘New Europe’ or ‘Central Europe’ (Garton Ash 1999; Kundera 1984), attempting to dissociate from the East risks reproducing the teleological horizon of Europe and lapsing into eurocentrism. They merely shift, as scholars have observed, the boundary of the ‘backward East’ further to the East, locating the Other elsewhere (Kuus 2004; Melegh 2006), rather than breaking with it. It is also important to think of the East – like the North and the South – not primarily in geographical terms, but as an ontological and epistemological category, so as not to risk collapsing it with a delimited world region.

‘The East’ is, of course, a polysemic, malleable term. In its various uses, it has extended from Eastern Europe and Russia to Japan and China, to what is sometimes known as the Middle East, including Turkey (Goody 1996; Mahbubani 2008; Mignolo 2014b; Neumann 1999; Said 1978; Zarakol 2011). That it usually serves as the Other of a thus affirmed West is one of its persistent features. This paper develops its argument in thinking from one of those multiple Easts: those societies in the former Second World that experienced the domino collapse of socialism between 1989 and 1992. But if Eastness is a predicament of semi-alterity, the notion of the Global East should not remain limited to those societies. It should, indeed, be relevant for all those that are neither hither nor thither.

It is its malleability that serves a concept such as the Global East well, suggesting not clear-cut boundaries and fixed territories but topological links and blurred zones. The East is always elsewhere: when I ask in France, the East is in Germany; when I ask in Germany, the East is in East Germany; when I ask in East Germany, the East is in Poland; when I ask in Poland, the East is in Ukraine, … a continuous displacement of signifieds attached to the signifier of the East. ‘The East’ can thus be thought of as a floating signifier, a signifier without a fixed signified. A signifier that says more about the person using it than about the object thus denoted. That feature makes it amenable to serve for the political project of re-inscribing the East. With Laclau (2005, chap. 5), floating signifiers enable the articulation of political demands, since they are able to accommodate a diversity of meanings (in Laclau’s parlance, they link political demands in a chain of equivalence). By virtue of not having any fixed meaning themselves, they lend themselves to the inscription of meaning.

It is that quality of malleability, inclusiveness and pointing beyond fixed territories that is much less present in concepts such as ‘Eurasia’, which has become popular to denominate large parts of the former Soviet Union (Russia and Central Asia in particular) (Grant 2012; Hann et al. 2016). Eurasia is very much a territorial concept and one, at that, with a problematic meaning tied to the ideology of Eurasianism that has experienced a revival in Russia for a while now (Suslov and Bassin 2016). Eurasianism has given nationalist and extremist forces in Russia moral and pseudo-scientific legitimation to pursue an ideology of empire. Laruelle (2016, 136) sums up this problematic aspect: ‘[Eurasia] expresses, conveniently and in a rather intuitive way, the historical space of Russia and its “peripheries,” and a certain, fast-moving geopolitical reality. … In countries such as the Baltic states and Ukraine, being studied academically as part of a Eurasian Department raises concerns’. Smith and Richardson (2017, 4–5) call Eurasia a ‘myth’: ‘[an] incoherent mess of spaces. … We find a Eurasia of myriad forms … defined by inconsistencies and incoherence’.

If we accept that proposition of polyphony and inconsistency, then we perhaps best think of the East as a strategic essentialism (Spivak 1988b): a political practice to mobilise heterogeneous marginalised groups to band together under a common banner for an emancipatory political project. Strategic essentialisms put aside differences for a while to articulate political demands vis-à-vis a hegemonic discourse. Many things can be ‘political’ in this context: the right of recognition, the production of what counts as valid knowledge, the freedom from discrimination. Such strategic essentialisms have been important tactics in feminism (Rose 1993), post-colonialism (Spivak 1993) and, more recently, in the rally for the Global South (Comaroff and Comaroff 2011; Parnell and Robinson 2012) to voice demands.

The case of the Global South, also a strategic essentialism, is particularly instructive here, as it also revolves around a marginalised but highly heterogeneous category and involved reclaiming a paternalistic concept. In fact, when the term ‘South’ (then still without the ‘global’) emerged in debates in the 1970s, it was a poorly disguised replacement for ‘developing countries’ that signalled the paternalistic responsibility of the North to rectify inequalities and ‘save’ the poor South. It was more regressive than the term ‘Third World’, which had become a political project owned by the South, marked by the Bandung Conference of 1955 (Dirlik 2007; Prashad 2013).

The recent push to start theorising from the South and dislodge the telos of modernity (associated with the Global North) has re-inscribed the meaning of the South (Chakrabarty 2007; Comaroff and Comaroff 2011; Robinson 2006): not just can and must the South be a source of theorising in its own right, but these theoretical insights speak to theorising in the North.^6^

The argument is perhaps most pronounced in Comaroff and Comaroff (2011), who see the North, with its perennial crises, instability and insecurity, austerity and social and ethnic cleavages, as evolving Southward and thus, in turn, the South as frontrunner of a theorisation of this novel condition. ‘The so-called “New Normal” of the North is replaying the recent past of the South’ (Comaroff and Comaroff 2012, 123).^7^ The North as playing catch-up with the South: what a refreshing way of turning the world on its head. Although the Comaroffs’ argument rests mostly on the deleterious global rule of neoliberalism – a rule that seems increasingly shaky with recent nationalist tendencies – the thrust of the argument is clear: the South has something to tell, not just to itself but also to the North.

The South’s push for emancipation may be a model for the East in its political thrust to decolonise the production of knowledge and put itself back on the map. This putting back on the map, however, cannot happen without thinking of the East as part of the global: as a Global East.

## A Global East: Following the Bevelled Drinking Glass

**Figure 1.**
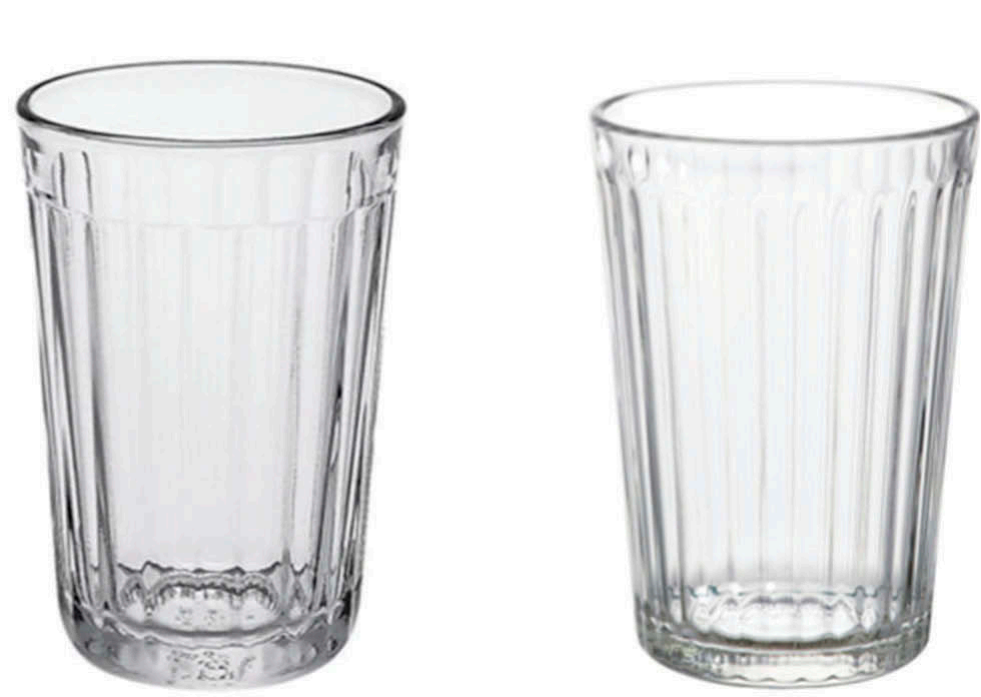
Can you spot the difference? The Soviet *granyonyi stakan* (left) and IKEA’s contemporary re-interpretation called *Vardagen* (right).

Let us try and think of a Global East that is not cut off from the world, frozen in space and time. Let us think of the Global East through the bevelled drinking glass (Figure 1), an IKEA staple. The bevelled drinking glass is ubiquitous around the world. It stands on the tables of presidents and students. I have it next to me here on my desk. And I bet you have had it in your mouth, too. There are no official figures, but I reckon, based on my casual observation of people’s cupboards in different parts of the world, that total sales have reached several billions. No wonder, at not even 70 cents per unit, the bevelled drinking glass is priced competitively and survives almost any attempt, intentional or not, to destroy it. Although most of us think of it as an IKEA product, the bevelled is a materialisation of the East that has gone global. IKEA’s design does not conceal its inspiration from a Soviet design classic, Vera Mukhina’s *granyonyi stakan* (гранёный стакан), a staple of Soviet glassware since at least 1943 (Idov 2011, 78).

One could be tempted to read of the bevelled drinking glass as yet another story of the ‘banal cosmopolitanism of consumer culture’ (Featherstone 2006, 390) and of global corporations riffing on (or ripping off?) vernacular knowledge. But it is quite a bit more than that. For one thing, the *granyonyi stakan* already had its first success story in the East, where, after World War II, five or six hundred million glasses were produced annually (Idov 2011, 80). So the glass should rather be read as a global consumer product designed in the East, copied in the West. This represents a rare reversal of the normal order of things in a world where design seems to be a prerogative of the creative centres of the Global North, encapsulated in the ubiquitous ‘designed in California, assembled in China’ on Apple products.

**Figure 2.**
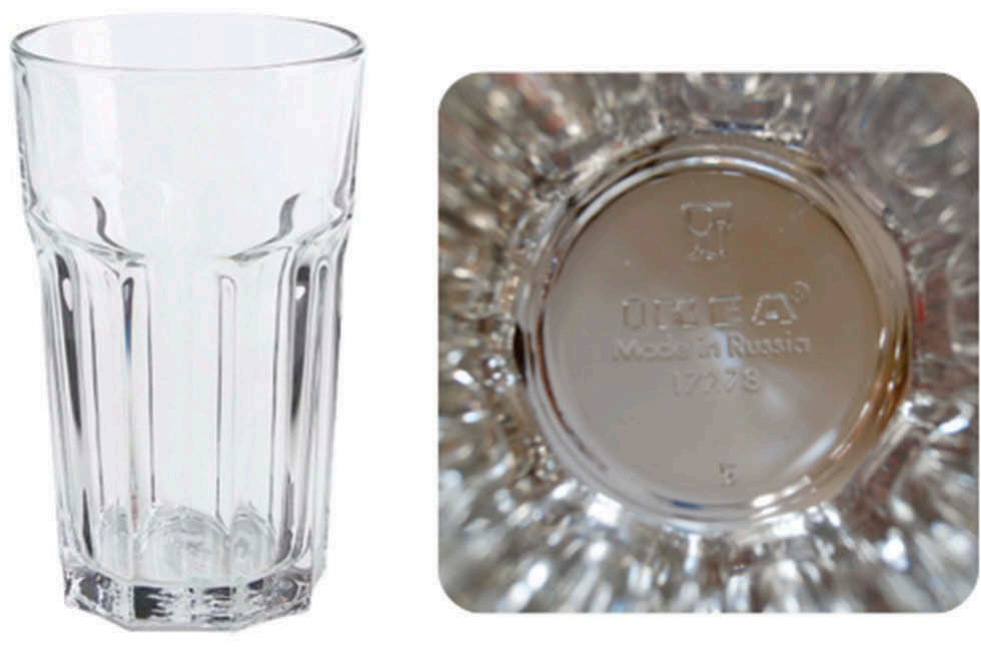
Made in the Global East: IKEA’s flagship glass Pokal.

But the bevelled drinking glass also has a production story to it. Much of IKEA’s glassware is made in the Global East. Cheap inputs (primarily energy) and expertise in glass-making mean that there is a competitive edge. IKEA produced its best-selling glass product, Pokal (see Figure 2), in Russia for a long time, before switching to Bulgaria, presumably due to the country’s still low production costs but membership of the EU, which made export easier.

The story of the bevelled drinking glass is that of an interstitial position of the Global East as both on the contributing and on the receiving end of globalisation. ‘Designed in the Global East, made in the Global East’ – this formula expresses the duality of the Global East in globalisation. But it also drives home another important point: that the East is entangled in global *relations*. It is bound up with the world, not withdrawn. This seems like an obvious point, but the East, as we have seen, is often conceived of as the very opposite: as fallen out of space and time.

Viewing the Global East as relational implies a topological concept of the East (Shields 2012; for the East specifically see Rogers 2010; Tuvikene 2016), where anywhere can become part of the East if it is tied into the right relations. The material presence of the IKEA glass on my desk right here ties me to the Global East; so does the edition of *Made in Russia* lying right next to it. In other words, the challenge of the Global East is to think in topological fashion: to think along relations that draw the far away close and bind together things that are not adjoining (Mol and Law 1994). The Global East – as relation – can be anywhere. Asking ‘where is the Global East?’ is therefore the wrong question, because it points us to territories. We would do better to ask ‘what is the Global East?’, directing us to relations.

So the point of a relational notion of the East is precisely not to limit it to the territories of the former Second World. When Soviet writer Ilya Ehrenburg wrote that Berlin was the stepmother of Russian cities, he was hinting at the trans-local relation of the East. Ehrenburg was one of the hundreds of thousands Russian émigrés after the October Revolution who had found an *Ersatz* home in Berlin and made it, in the words of Karl Schlögel, a surrogate capital (*Ersatzhauptstadt*) (Schlögel 2007). But one does not need to go back one hundred years to Berlin’s Charlottengrad to find the Global East everywhere: in the Russian-speaking communities in New York’s Little Odessa (Miyares 1998) and Cyprus’s Limmasol, in London’s East European communities (Neumann 2015), in Ukraine’s transnational evangelicals (Wanner 2007). It is there in Russian interference in elections in the United States, in ‘mobile mothers’ that shuttle between Moldova and Istanbul (Keough 2016), in the connections between the oil-rich regimes in Central Asia and the Persian Gulf (Koch 2016), in the Republic of Georgia’s global promotion as a poster child for post-Soviet reform (Schueth 2011) – and in the story of consumer products such as the *granyonyi stakan*.

In a way, the most interesting part of the East is perhaps not its core, but its extensions and trading zones. Following these extensions also opens up new avenues for comparison of countries, phenomena and places that many would consider too different to be comparable but which, for that very reason, can produce revealing insights. Comparing the neo-patrimonial garrison states of the United States and Russia, the built modernism of Tashkent and Brasília and the evangelicals of Ukraine and Nigeria has an important epistemological function: it establishes the East not as fundamentally different and guards against its exoticisation as Other. It decentres the West and universal knowledge claims emerging from there (Robinson 2016; Sidaway 2013).^8^

The focus on relations reveals the affinities of the concept of the Global East to a third wave of area studies, much shaped by processes of globalisation, connection and mobilities, and the engagement with social and cultural theory (Middell 2013; Mielke and Hornidge 2016; Sidaway et al. 2016). This third wave, self-reflexive as it is, critically assesses previous assumptions of areas as bounded and the colonial production of knowledge about areas from the centre, seeking to push towards an analysis of global connections and a decentring of knowledge production. It treads a precarious path between dismissing localised expertise out of hand altogether in favour of disembedded global studies (Koch 2016, 650) and reifying regions as self-contained entities (van Schendel 2002). For the Global East, the important realisation is this: knowledge from places remains important, without having to be attributed to a particular region as an epistemological frame. In other words, where something happens makes a difference, but it does not make all the difference. Places may create an affordance, a specific possibility for action, that may or may not be actualised. In Chari’s (2016, 792) phrasing, we need to commit to ‘being-with in a world of simultaneous interconnection and ontological difference’. This asks us to cultivate the Global East through a global sense of place: not self-enclosed and defensive but outward looking, without ever losing whatever constitutes its specificities (Massey 1991).

## Conclusion: Theorising with the Global East

In thinking of the world as divided into a Global North and a Global South, the East has ended up in some sort of netherworld. Its interstitial position – not quite rich, but neither quite poor; not just colony, but neither just coloniser – has made it difficult to categorise. Cast as backward and closed-off, scholarship and public discourse have had a tendency to consider the East as somehow separate and distant from the world, not having much to contribute to it.

This article has made the case for embracing the East as a Global East: to reclaim a place *and* a voice for it in the world. This involves thinking of the East as a strategic essentialism that allows both re-establishing it as a pertinent preoccupation of scholarship and re-inscribing it with new meaning. The East as Global East means placing the East right in the middle of the world. If we see the Global East as bound up with multiple other places, it becomes harder to leave it at the sidelines of theorising. Neither North nor South, it helps us avoid hemispheric binaries of rich and poor, powerful and powerless when thinking of the global. Engaging in global comparative research guards against isolation of the East by making its multiple experiences – of empire, globalisation, neoliberal reform, nationalist populism, political resistance, asymmetric wars – speak to similar debates elsewhere.

Thinking of a Global East is therefore also a political project. Not only does it write against the demi-othering of the East in the North and the silence about the East in the South, thus seeking to make the experiences of people in the East count and dislodge eurocentrism, but it also affirms the East as an open place, reaching out rather than withdrawn. As we see a surge of nationalist populism in the North, South and East that seeks to wall off countries rather than build bridges, asserting openness becomes an important political statement. With the supposed heartlands of liberal democracy in Britain and the United States in turmoil, this political project may come at an opportune time, when the differences between North and South, East and West become increasingly blurred and established hierarchies are called into question.

While this paper has theorised the Global East from the vantage point of the former socialist societies of the Second World, the condition of Eastness – a demi-otherness that hovers between North and South – extends much beyond. What about South Korea, Turkey or the ominous Middle East? Thinking with the East means drawing out the silences of the hemispheric North–South divisions, working not so much from its margins as from its interstices. The idea is not to resurrect another binary – that of West–East – but to destabilise the binary geopolitical imagination through the introduction of a *tertium quid*.

In embracing the Global East as *tertium quid*, we would therefore do well to also embrace its liminal position, its semi-alterity. The traditional response has been to think of this interstitial position as something to get rid off or transition from, in the move from periphery to core. But why do we not think of this liminality as a strength? Why do we not utilise the resources it offers to address uncertainties, unpredictabilities and improvisational tactics? Thinking from between North and South would then mean thinking about ambiguity and ephemerality – not just for the East, but also for the North and South.

In this way, embracing its liminality inscribes the Global East not just into multiple unfurling debates around the geopolitics of knowledge (Mignolo 2002) – a third wave of area studies that interrogates the politics of representation and foregrounds transnational links (Sidaway et al. 2016) and theorising from the South (Connell 2007) – it also invites to tease out what thinking with the East means for conceptualising the paradoxes and uncertainties that mark globalising societies and that have gained so much traction in scholarship in the past decades (Bauman 2006; Prigogine 1996; Žižek 2011).

Theorising *about* the Global East will therefore not be enough. Re-inscribing the Global East cannot happen without turning the tables on knowledge production about it. In so doing, we need to turn the East from an object of area studies into a subject, or indeed a method – a ‘means of transforming knowledge production’ (Chen 2010, 216). It matters whence this re-inscription takes place. The recent upsurge of scholarship from the Global East leaves little doubt that the time has come to theorise not just about, but *with* the Global East.

## Notes

1. In accordance with common usage, I write ‘communism’ when referring to the ideology and ‘socialism’ when referring to the socio-political realities.

2. Molvanîa is a fictional country, portrayed in a parody of a guidebook that plays on Western stereotypes of the East (Cilauro, Gleisner and Sitch 2004). See also Malcolm Bradbury’s invention of the fictional East European country Slaka (Bradbury 1986).

3. The French original is much pithier than the translation: ‘tout est prévu, trouvé, prouvé, exploité’ (Fanon 1952, 97).

4. Wallerstein proposes a three-tier structure of stratification into core, semi-periphery and periphery, which results from two forms of economic processes: core processes of high wages, advanced technology and a diversified production mix and peripheral processes that involve low wages, more rudimentary technology and a simple production mix. His intermediate category of the semi-periphery is not just an economic mix of the two, but, more importantly, has a political function: it provides political stability by taking the edge off the polarisation between core and periphery. ‘One might make a good case that the world-economy as an economy would function every bit as well without a semiperiphery. But it would be far less *politically* stable’ (Wallerstein 1979, 23). According to Wallerstein (69), the two characteristics of semi-peripheral countries are protectionist policies (‘home market for home products’) and the politicisation of economic decisions.

5. The perspective of post-socialism, an attempt to make sense of the East after the socialist collapse (e.g. Hann 2001), was a product of those circuits, lodged in Anglo-American scholarship. Scholars in the East have critiqued it for being ‘an orientalising concept through which western anthropologists constructed postcommunist Europe’ (Červinková 2012, 159).

6. This is also true for the recent push to theorise from Asia in the ‘Asian century’; see for example Kuan-Hsing Chen’s (2010) *Asia as Method* and Kishore Mahbubani’s (2008) *The New Asian Hemisphere.*

7. The frequent invocation of the differences between North and South should not elide that literature has started to look at the relations that cut across North and South and blur boundaries (e.g. Caison and Vormann 2015; Roy and Crane 2015).

8. Recent literature on the Global North and South has stressed these topological relationships of the North in the South (e.g. gated communities) and the South in the North (e.g. poverty) (e.g. Miraftab 2009; Roy and Crane 2015).
